# Targeting Bcl-2 Proteins in Acute Myeloid Leukemia

**DOI:** 10.3389/fonc.2020.584974

**Published:** 2020-11-05

**Authors:** Yunxiong Wei, Yaqing Cao, Rui Sun, Lin Cheng, Xia Xiong, Xin Jin, Xiaoyuan He, Wenyi Lu, Mingfeng Zhao

**Affiliations:** ^1^ The First Central Clinical College of Tianjin Medical University, Tianjin, China; ^2^ Nankai University School of Medicine, Tianjin, China; ^3^ Department of Hematology, Tianjin First Central Hospital, Tianjin, China

**Keywords:** B cell lymphoma 2, AML—acute myeloid leukemia, venetoclax (ABT-199), intrinsic apoptosis, Bcl-2 protein

## Abstract

B cell lymphoma 2 (BCL-2) family proteins play an important role in intrinsic apoptosis. Overexpression of BCL-2 proteins in acute myeloid leukemia can circumvent resistance to apoptosis and chemotherapy. Considering this effect, the exploration of anti-apoptotic BCL-2 inhibitors is considered to have tremendous potential for the discovery of novel pharmacological modulators in cancer. This review outlines the impact of BCL-2 family proteins on intrinsic apoptosis and the development of acute myeloid leukemia (AML). Furthermore, we will also review the new combination therapy with venetoclax that overcomes resistance to venetoclax and discuss biomarkers of treatment response identified in early-phase clinical trials.

## Introduction

Acute myeloid leukemia (AML) is one of the most common hematological malignancies in adults, with a median age at diagnosis of 68 (1). For the past 30 years, the main treatment has been chemotherapy alone or combined with hematopoietic stem cell transplantation (HSCT). In 2017, there was a breakthrough, and several new drugs, including venetoclax and selective B cell lymphoma-2 (BCL-2) inhibitors, seemed to reshape the therapeutic landscape of AML (2). The BCL-2 family of proteins plays a significant role in the intrinsic apoptotic pathway. They are also critical for cell survival and are overexpressed in many tumors, including AML. In addition, aberrant overexpression of BCL-2 family proteins causes resistance to chemotherapy and is associated with a poor prognosis (3–7). Recently, BCL-2 inhibitors, such as venetoclax and novel MCL-1 inhibitors, have shown anti-leukemic activity in preclinical AML models. In 2018, venetoclax in combination with azacitidine, decitabine, or low-dose cytarabine (LDAC) was approved for use in untreated patients with AML aged 75 years or older or patients with comorbidities that preclude the use of intensive induction chemotherapy (8). Here, we review how they regulate apoptosis and how the targeting of BCL-2 has developed in AML. Furthermore, we summarize the mechanism of resistance to venetoclax and provide some potential solutions.

## Apoptosis and the BCL-2 Family

**Figure 1:**
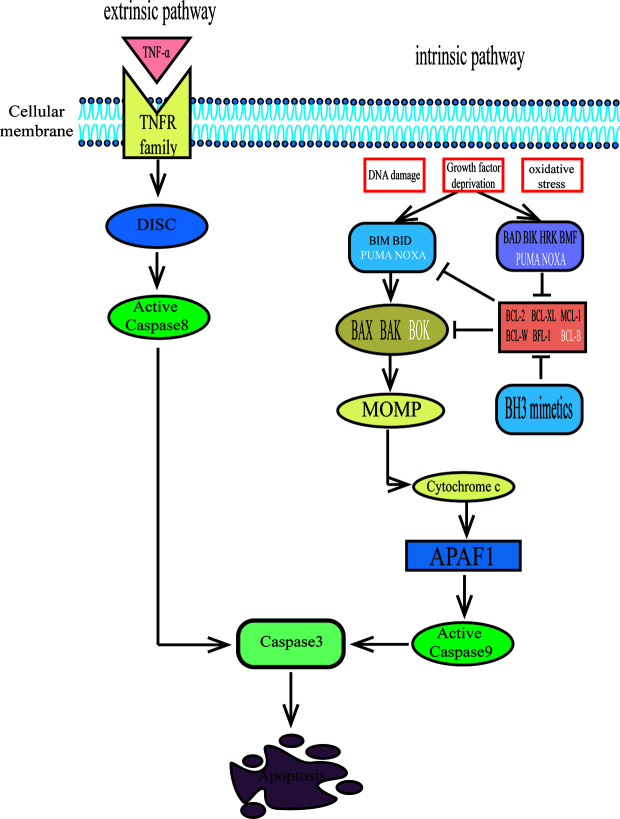
The extrinsic and intrinsic pathways to apoptosis. The extrinsic pathway of apoptosis is activated when certain death receptor ligands of the tumour necrosis factor (TNF) family (such as TNF) engage their cognate death receptors on the plasma membrane, leading to caspase-8 activation via the death-inducing signalling complex (DISC), which results in apoptosis. The intrinsic pathway of apoptosis is activated by cellular stresses (such as DNA damage, growth factor deprivation or oxidative stress) and is regulated by BCL-2 family proteins. BH3 proteins bind to and activate pro-apoptotic proteins BAX, BAK and possibly BOK, which oligomerize in the mitochondrial membranes and release cytochrome c, then interact with apoptosis protease-activating factor 1(APAF1) and initiate caspase activation that results in apoptosis. Futhermore, BH3-only activator proteins (BID, BIM, Puma and NOXA) directly bind to interact with BAX and BAK to promote mitochondrial outer membrane permeabilization (MOMP). While the BH3-only sensitizer proteins (BAD, BIK, HRK, BMF, PUMA, NOXA) bind to anti-apoptotic proteins with higher affinity, freeing the activator proteins from the BH3 binding pockets in the anti-apoptotic proteins and executing cell death by binding to BAX or BAK. The two pathways converge at activation of the effector caspase-3.

There are two main pathways for apoptosis: the intrinsic pathway and the extrinsic pathway (9). The extrinsic apoptotic pathway is initiated by the interaction of an extracellular ligand and tumor necrosis factor (TNF) family death receptors. Then, procaspase-8 is activated by the binding of the death-inducing signaling complex (DISC) to a series of adaptors, and activated caspase-8 further initiates the caspase-3 cascade, which eventually leads to cell death *via* apoptosis (10, 11) (**Figure 1**). The intrinsic pathway is initiated by internal cellular stress, such as DNA damage, growth factor deprivation, and oxidative stress. These alterations then lead to mitochondrial depolarization, which allows the release of cytochrome c, which is the hallmark of the intrinsic apoptotic pathway. Cytochrome c binds to apoptosis protease-activating factor 1 (APAF1) and procaspase-9, forming an intracellular “apoptosome” that can activate caspase-9. Then, active caspase-9 leads to executioner caspase-3 activation (11) (**Figure 1**). The BCL-2 family of proteins regulates the intrinsic apoptotic pathway by controlling mitochondrial outer membrane permeabilization (MOMP) (12).

The BCL-2 gene, whose transcription can be upregulated, was first discovered as a part of t(14;18) chromosomal translocation in follicular lymphoma and diffuse large B cell lymphoma (13, 14). It was first recognized as a classical growth-driving oncogene, but later proved that BCL-2 promotes malignant cell survival by attenuating apoptosis (15, 16). The BCL-2 family consists of more than 20 proteins, which may be functionally classified as either anti-apoptotic or pro-apoptotic proteins (17). The anti-apoptotic BCL-2 proteins, which include four BCL-2 homology domains (BH1–4), include BCL-2, BCL-XL (BCL-2-like protein 1), MCL-1 (induced myeloid leukemia cell differentiation protein MCL-1), BCL-W (Bcl-2-like protein 2), BFL-1/A1 (BCL-2-related protein A1), and possibly BCL-B (Bcl-2-like protein 10). The pro-apoptotic proteins can be divided into effector proteins and BH3-only proteins. The former group contains three BH domains and includes BAX (BCL-2 associated X protein), BAK (BCL-2 antagonist killer), and possibly BOK (BCL-2-related ovarian killer). The latter group possesses only a single BH3 domain and includes BID (BH3-interacting domain death antagonist), BIK (BCL2 interacting killer), BIM (BCL-22L11; BCL2-interacting mediator of cell death), BAD (BCL2 antagonist of cell death), BMF (BCL-2 modifying factor), HRK (activator of apoptosis hara-kiri), PUMA (p53 upregulated modulator of apoptosis; also called BBC3 [BCL2 binding component 3]), and NOXA (PMA induced protein 1; also called PMAIP1 [phorbol-12-myristate-13-acetate-induced protein 1]) (11, 17, 18). Once BAX and BAK are activated, they oligomerize and form pores to induce MOMP, releasing cytochrome c from mitochondria and finally inducing cell apoptosis (19). The anti-apoptotic BCL-2 proteins can protect cells from apoptosis by directly binding to BAX/BAK or antagonizing pro-apoptotic BH3-only proteins. Furthermore, the BH3-only proteins can be subdivided into activator and sensitizer proteins. The activator proteins, which include BID, BIM, Puma, and NOXA, directly bind to and interact with BAX and BAK to promote MOMP. The sensitizer proteins bind to anti-apoptotic proteins with higher affinity, freeing the activator proteins from the BH3 binding pockets in the anti-apoptotic proteins and executing cell death by binding to BAX or BAK (20, 21) (**Figure 1**).

**Figure 2:**
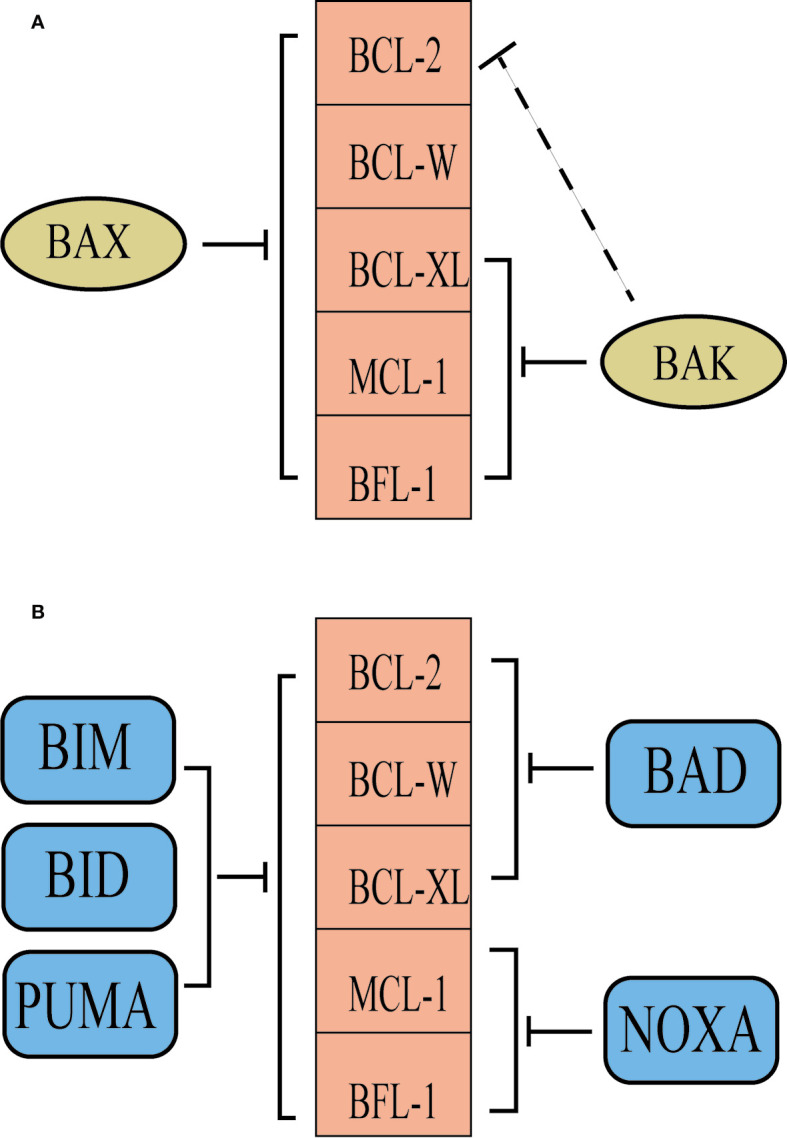
The selectivity of BCL-2 family proteins. **(A)** BAK is inhibited predominantly by BCL-XL, MCL-1 and BFL-1, while BCL-2 contributes in some situations. However, BAX is probably inhibited by all of the pro-survival proteins. **(B)** Some BH3-only proteins, such as BAD and NOXA, are selective for subsets of their anti-apoptotic relatives, whereas other BH3-only proteins, particularly BIM, BID and PUMA, probably neutralize all of the anti-apoptotic proteins.

Owing to subtle differences in their BH3 domains and in the grooves of the anti-apoptotic proteins, the various BCL-2 family proteins have differential specificity of binding to one another. For example, BAX and BAK have high affinities for BCL-XL, and BAK has higher affinities for MCL-1, as do BAX and BCL-2 (22). Interestingly, some BH3-only proteins, such as BAD and NOXA, are selective for subsets of their anti-apoptotic relatives, whereas other BH3-only proteins, particularly BIM, BID, and PUMA, probably neutralize all of the anti-apoptotic proteins (23) (**Figure 2**). In healthy cells, anti-apoptotic proteins and pro-apoptotic proteins maintain a delicate balance, while cancer cells overexpress different anti-apoptotic proteins to facilitates prolonged tumor cell survival (24). A good knowledge of the roles of BCL-2 family proteins in promoting tumorigenesis has contributed to the development of numerous novel drugs targeting aberrant apoptotic pathways in cancer. Thus, targeting BCL-2 family proteins may be a prominent strategy for cancer therapy.

## BCL-2 in AML

A number of early studies showed that BCL-2 was overexpressed in CD34+ AML cells and was associated with poor prognosis and resistance to chemotherapy (25–27). Overexpression of MCL-1 and BCL-XL also confers chemotherapy resistance in AML (4, 28, 29). Thus, antisense oligonucleotides targeting BCL-2 were explored and decreased the number of leukemia cells *in vitro* (30). A better understanding of the intrinsic apoptosis pathway has contributed to the focus on the development of novel small molecules that mimic the BH3 domain found in all pro-apoptotic BCL-2 family proteins. These drugs mimic the action of certain pro-apoptotic BH3-only proteins by binding directly to the BH3-binding domains of anti-apoptotic molecules, thereby displacing native BH3-only proteins and thus inducing apoptosis (**Figure 1**). These protein-protein interaction inhibitors that directly activate apoptosis represented a milestone event in medicinal chemistry, as well as in the cell death field. With the inspired success in B cell lymphoid malignancies, these new BH3 mimetics have also been used in AML.

## Early Attempts to Target BCL-2 in AML

### Oblimersen

Oblimersen, which can target the first six codons of the human BCL-2 mRNA, is an 18-mer phosphorothioate Bcl-2 antisense oligonucleotide. It can increase cancer cell apoptosis and overcome chemotherapy resistance by binding to BCL-2 mRNA, thus downregulating BCL-2 protein expression (30, 31). The first use in a clinical trial of AML was a phase I study in relapsed or refractory acute leukemia in which patients received a combination with fludarabine, cytarabine, and granulocyte colony-stimulating factor (FLAG) salvage chemotherapy (32). Seventeen patients with relapsed and refractory AML and 3 patients with acute lymphoblastic leukemia (ALL) were recruited. Five of 17 (29%) achieved complete response (CR), and 2 patients achieved complete response with incomplete blood count recovery (CRi) (11%), yielding an objective response of 41%. Furthermore, no dose-limiting toxicity was observed in any of the cohorts examined. Another phase 1 trial conducted by the same group combined two different doses of oblimersen with chemotherapy in untreated older patients with AML (33). In this trial, 14 of 29 (48%) patients achieved CR, with no significant increase compared with the previously reported remission rates of 40% in the absence of oblimersen. The side effects were similar to those expected with chemotherapy alone and were not dose limiting at either dose level, which proved that the drug was safe and tolerable. The preclinical and early clinical data led the Cancer and Leukemia Group B (CALGB) to conduct a large phase III trial in which older adults with newly diagnosed AML were randomized to standard induction chemotherapy with or without oblimersen (34). Regrettably, there were no differences observed in CR rates (48% vs 52%; p=0.75) or overall survival rates (OS at 1 year 36% vs 40%; p=0.83), which may lead to no further trials of this drug in AML. However, the failure of this trial did not dispel the exploration of BCL-2 inhibitors in AML.

### Obatoclax (GX15-070)

Obatoclax is a pan-Bcl-2 antagonist and was the first BH3 mimetic used in clinical trials of AML. It can bind to the BH3 domain of BCL-2 (as well as those of BCL-XL, MCL-1, BCL-w, A1, and BCL-b) (35) and then prevent the anti-apoptotic proteins from sequestering pro-apoptotic BH3-only proteins. Obatoclax potently induced apoptosis and decreased leukemia cell proliferation in AML cell lines and primary AML samples (36). In a phase I trial of 44 patients with advanced hematological malignancies, which included 25 AML, 14 myelodysplasia (MDS), 4 chronic lymphocytic leukemia (CLL), and 1 ALL, patients showed good tolerance but modest efficacy in the clinic (37). Only one patient with AML with mixed lineage leukemia t(9;11) rearrangement achieved complete remission. In addition, obatoclax had neurological side effects, which further limited its clinical development. In addition, another phase I/II study of obatoclax in older patients with previously untreated AML showed similar results in that obatoclax did not seem to be associated with an objective response (38). The failure of this drug may encourage a future study on its combination with other new drugs, such as histone deacetylase inhibitors or sorafenib (39, 40). Furthermore, the insolubility of this compound also limits its function, so new BH3 mimetics should be explored.

## Dual BCL-2/BCL-XL Inhibitors

### ABT-737

ABT-737 is a BH3 mimetic with high potency activity against BCL-2, BCL-XL, BCL-W, and, to a lesser extent, MCL-1. It was discovered as the first high-affinity inhibitor of BCL-2 family proteins by using nuclear magnetic resonance (NMR)-based screening (41). In a preclinical study, ABT-737, which can effectively trigger Bax/Bak-mediated apoptosis, induced AML cell apoptosis *in vitro*, and the same activity was demonstrated in a murine xenograft model *in vivo* (42–44). High expression of MCL-1 and phosphorylation of BCL-2 are associated with resistance to ABT-737, as they lead to a reduction in MCL-1. Thus, targeting these changes may be a potential way to enhance ATB-737 response. For example, ABT-737 could be combined with pan-Bcl-2 family inhibitors, MEK inhibitors, or PI3K/mTOR inhibitors (45–47). However, owing to its poor oral bioavailability and solubility in water, further studies on this drug are likely to be limited.

### Navitoclax (ABT-263)

Navitoclax, which is a derivative of ABT-737, is an improved orally bioavailable BH3 mimetic with high affinity for BCL-2, BCL-XL, and BCL-w and substantially lower affinity for MCL-1 than ABT-737 (48). Navitoclax also showed efficacy in AML preclinical studies (49–51). In early clinical trials, it has shown activity in CLL and small-cell lung cancer (52, 53). However, it can cause thrombocytopenia due to its effect on BCL-XL (54–56). This on-target toxicity of navitoclax has undoubtedly limited its further development, which prompted AbbVie’s development of venetoclax (ABT-199).

## Selective BCL-2 Inhibitors

### Venetoclax

Venetoclax is a highly selective small molecule BH3 mimetic with subnanomolar affinity (Ki<0.01 nM) for BCL-2 and nanomolar affinity (Ki<245 nM) for BCL-XL. Based on this feature, it circumvents significant thrombocytopenia due to concomitant inhibition of BCL-XL, making it clinically available for the treatment of AML, a disease typically associated with thrombocytopenia (57, 58). In preclinical studies, venetoclax induced rapid cell death in AML cell lines and primary patient samples *in vitro* and in a mouse xenograft model *in vivo* (58). In the study of this drug, mitochondrial BH3 profiling could be used as a biomarker for predicting the response of AML primary cells to venetoclax *in vitro* and in a patient-derived xenograft model *in vivo*. Considering the preclinical data for ABT-767 and navitoclax and the efficiency and safety of venetoclax in CLL, venetoclax was immediately moved into clinical trials.

## Selective BCL-XL Inhibitors

To date, many studies on BCL-XL inhibitors have focused on solid tumors (such as breast cancer, non-SCLC, ovarian cancer, colorectal cancer, and multiple myeloma) because solid tumors rely on BCL-XL for their survival. Selective BCL-XL inhibitors include WEHI-539, A-1155463, and A-1331852 (59–63), and further study may focus on the use of these inhibitors in AML.

## Selective MCL-1 Inhibitors

MCL-1 has been shown to play a significant role in promoting cell survival in AML cell lines (64), and its overexpression in tumor cells may be associated with resistance to radiotherapy, chemotherapy, and BH3-mimetics targeting BCL-2/BCL-XL (65–67). Overexpression of MCL-1 has been identified in many primary AML cells and as a major factor in the development of resistance to venetoclax (68, 69). Thus, it led to the development of MCL-1 inhibitors. There are a number of MCL-1 inhibitors, and we reviewed several MCL-1 inhibitors that have entered clinical trials.

To date, S63845 has been identified as the most promising selective MCL-1 inhibitor. It showed low nanomolar cytotoxic activity in multiple hematological cancer-derived cell lines *in vitro* and potent efficacy *in vivo* in preclinical mouse models of diverse hematological malignancies (70). A side-by-side comparison of BH3 mimetics showed that MCL-1 may be a more potent therapeutic target than BCL-2 in AML (71). Therefore, the utilization of S63845 should be the first line of choice in future clinical trials.

Very little information on S64315 (MIK665) has been disclosed, with two clinical trials under investigation in patients with AML or myelodysplastic syndrome (NCT02979366, NCT03672695) (72).

AZD5991, which has high selectivity and affinity for MCL-1, has been shown to cause an effective apoptotic response in AML cell lines at a low nanomolar range. Furthermore, AZD5991 binds directly to the MCL-1 and BAK interaction and was shown to have potent antitumor activity *in vivo*, as demonstrated by high tumor regression in an AML xenograft model. Based on these promising data, a phase I clinical trial is undergoing relapsed or refractory hematological malignancies (NCT03218683) (73, 74).

AMG176 is a first-in-class selective MCL-1 inhibitor that is being studied in humans. It targets the BH3-binding groove of MCL-1 and thus frees BAX, resulting in activation of the intrinsic apoptotic pathway. In preclinical studies, it showed potent effects on AML cell lines, xenograft models, and primary patient samples. Based on this background, AMG176 is under several clinical investigations for AML (NTC02675452, NCT03797261) (75, 76).

## Clinical Uses of Venetoclax

### Single-Agent Venetoclax in Patients With Relapsed/Refractory AML

The first clinical trial of venetoclax in AML was a phase 2 single-agent study in patients with relapsed/refractory disease or untreated patients ineligible for intensive chemotherapy (77). In this study, they recruited 32 patients, with a median age of 71 years, which consisted of 30 relapsed/refractory patients and 2 treatment-naïve patients that were ineligible for intensive chemotherapy. This study showed a modest overall response rate of 19% (6/32), with 6% of patients (2/32) achieving CR and 13% of patients (4/32) achieving CRi; another 19% of patients had anti-leukemic activity that did not meet the criteria for response. The median duration of remission was only 48 days, and the median time spent on the study was only 63.5 days. Most patients had high-risk features, including pre-existing myelodysplastic syndrome or myeloproliferative neoplasm (MDS or MPN, 41%), FLT3-ITD mutations (13%), and older age (median 71 years).

Interestingly, patients with IDH1/2 mutations had a stronger response, with 4 of 12 (33%) patients achieving CR/CRi. Moreover, another 2 patients with IDH mutations demonstrated anti-leukemic activity that did not meet formal criteria for response due to a lack of hematological recovery. This is consistent with preclinical studies, in which IDH1/2 mutant AML suppresses the activity of cytochrome c oxidase and lowers the mitochondrial threshold to trigger apoptosis upon BCL-2 inhibition (78). Another impressive discovery was that all 6 patients who achieved CR/CRi received prior hypomethylating agents, which may lead to the exploration of its combination with hypomethylating agents. The most common grade 3/4 adverse events (AEs) were febrile neutropenia (31%), hypokalemia (22%), and pneumonia (19%), and no tumor lysis syndrome (TLS) events were reported. Overall, this study showed that single-agent venetoclax demonstrated antitumor activity in AML with good tolerability.

### Venetoclax-Based Combinations in Treatment-Naïve Patients With AML

Preclinical models have proven a synergistic effect upon combining venetoclax and hypomethylating agents (HMAs) in AML cell lines and primary AML patient samples (79, 80). Moreover, studies have shown that azacitidine can reduce the protein levels of MCL-1, an anti-apoptotic protein that is associated with potential mechanism of resistance to venetoclax (81, 82). Based on these data, the combination of venetoclax with HMAs for the treatment of AML may be a promising therapeutic approach.

AbbVie launched a phase 1b clinical study (NCT02203773) that recruited 212 patients over the age of 60 years with treatment-naïve AML. They conducted a dose escalation and expansion study of venetoclax (83, 84). At first, a total of 45 patients were enrolled in the dose escalation, a dose of 400 mg of venetoclax was administered to 10 patients (4 in the azacitidine group, and 6 in the decitabine group), a dose of 800 mg of venetoclax was administered to 24 patients (12 each in the azacitidine and decitabine groups), and a dose of 1200 mg of venetoclax was administered to 11 patients (6 in the azacitidine group, and 5 in the decitabine group). Although the maximum tolerated dose was not reached in any group, the 1200 mg dose led to a high frequency of gastrointestinal AEs (nausea in 82% and diarrhea in 64% of patients) (83). As a result, the 400 mg and 800 mg dose cohorts for both azacitidine and decitabine were expanded, and 50 patients were added to each group. This study demonstrated a remarkable overall response rate of 68% and included CR and CRi rates of 37% and 30%, respectively. The median follow-up was 15.1 months, and the median OS for all groups was 17.5 months. In addition, the patients who received 400 mg venetoclax plus HMA achieved a remarkable CR/CRi rate of 73% (76% for azacitidine and 74% for decitabine), which revealed that the combinations with azacitidine or decitabine were not significantly different and that 400 mg of venetoclax might be a favorable dose (84, 85). Furthermore, patients with IDH1/2 mutations, FLT3 mutations, or NPM1 mutations had CR/CRi rates of 71%, 72%, and 91.5%, respectively. Even patients with poor risk features showed impressive responses, including those with high-risk cytogenetics (60% CR/CRi) and TP53 mutant patients (47% CR/CRi). The most common grade 3/4 adverse effects for all groups were thrombocytopenia (47%), febrile neutropenia (42%), and neutropenia (40%). No laboratory or clinical TLS was observed. Further studies explained how this treatment executed anti-leukemia activity. Leukemic stem cells (LSCs) rely on amino acid metabolism for oxidative phosphorylation and survival. Venetoclax with azacitidine was able to induce LSC toxicity *in vitro* by decreasing amino acid uptake, as confirmed by decreased α-ketoglutarate and increased succinate levels, suggesting inhibition of electron transport chain complex II. These metabolic perturbations suppress oxidative phosphorylation, which in turn efficiently and selectively targets LSCs (86, 87).

Similarly, preclinical studies demonstrated that cytarabine can boost venetoclax activity in AML by reducing MCL-1 levels (88, 89). An open-label, multicenter phase trial phase 1b/2 study of venetoclax in combination with LDAC recruited 82 patients over the age of 60 years (median age 74 years) with treatment-naïve AML (90). In the dose escalation phase, most patients needed dose interruption to permit blood count recovery and had a higher rate of hematological toxicity. Therefore, a 600 mg dose was recommended for the phase 2 study of the trial. The CR/CRi rate was 54% (CR, 26%; CRi, 28%). The median OS was 10.1 months, and the median duration of response was 8.1 months. Fifty-eight patients were treatment naïve; the CR/CRi rate for this group was 62%, which is similar to the 67% observed with venetoclax+ HMAs, whereas the group of patients who had prior HMA exposure only obtained a CR/CRi rate of 33%. Patients with mutations in NPM1 or IDH1/2 mutations had CR/CRi rates of 89% and 72%, respectively, which are higher than the average CR/CRi rate. However, patients with TP53 or FLT3 mutations had worse CR/CRi rates (30% and 44%, respectively). The most common grade 3/4 AEs were febrile neutropenia (42%), thrombocytopenia (38%), and neutropenia (27%). There were 2 cases of laboratory TLS and no evidence of clinical TLS.

In addition, a “real-world” report of venetoclax combined with azacitidine for the same patient populations at the same institution has been published (91). Thirty-three patients who received venetoclax + azacitidine off-trial were retrospectively analyzed and compared with 33 patients who received the same therapy on trial. The CR/CRi rate was 63.3% for off-trial patients who received treatment and 84.9% for trial patients, with a median OS periods of 381 days and 880 days, respectively. Although the responses and survival with venetoclax in real-world AML were inferior to those treated in a clinical trial, it still remained effective and tolerable compared with induction chemotherapy.

In summary, venetoclax in combination with HMAs or LDAC seemed to achieve a positive outcome in similar patient populations. On the basis of the above-described clinical data, two phase 3 trials comparing venetoclax + azacitidine/LDAC with azacitidine/LDAC alone are ongoing (NCT02993523, NCT03069352). The promising responses observed in these trials led to the FDA approval of venetoclax in combination with HMAs or LDAC for treatment-naïve AML patients who were at least 75 years old or ineligible for intensive induction chemotherapy because of comorbidities.

### Venetoclax-Based Combinations in Relapsed/Refractory Patients With AML

Less than 10% of patients with relapsed/refractory AML are cured with current standard chemotherapy. Allogeneic HSCT is the only realistic hope of a cure for these patients, but only a minority of patients consider this option (92). There is an urgent need to explore new strategies for these patients. Several retrospective studies of relapsed or refractory patients receiving venetoclax-based therapies have been reported. A single institution retrospectively analyzed 33 consecutive adults with relapsed/refractory AML in the real world, and the combination with HMAs achieved a CR/CRi rate of 33%, which included patients with no prior HMAs or allogeneic stem cell transplants (93). However, another series of 39 patients with relapsed/refractory AML achieved a dismal CR/CRi rate of 12% (8/39) (94). Moreover, in a series of 90 patients analyzed after either HMA treatment (51%) or allogeneic stem cell transplant (29%), the combination of venetoclax and HMAs achieved a CR/CRi rate of 46% in patients at a younger median age than the former study (95). Hence, the data revealed that venetoclax-based combinations may be a potential treatment strategy for patients with relapsed/refractory AML.

## Venetoclax Resistance and Combination Strategies to Overcome

Although numerous reports have shown that venetoclax-based therapies exert a promising effect on AML, they still remain partly resistant to venetoclax. It is necessary to explore how resistance evolves and how this can be overcome. The upregulation of MCL-1 protein in AML cells is one of the most well-known reasons for resistance to treatment with venetoclax (69). Therefore, targeting MCL-1 directly or indirectly is a new way to solve the resistance. Moreover, mitochondrial cristae structure may be another reason for resistance. Targeting mitochondrial architecture may provide a promising approach to circumvent it (96). Directly targeting MCL-1 has been introduced previously.

Here, we introduce several combinations that can reduce the MCL-1 level indirectly to circumvent the resistance. Indirect MCL-1 inhibitors include the following compounds: bromodomain extra-terminal protein inhibitors (BETis), which reduce MCL-1 and BCL-XL levels while increasing BIM levels and enhance the lethal effects of venetoclax on AML (97); cyclin-dependent kinase 9 (CDK9) inhibitors, which inhibit the transcription of MCL-1 (98); midostaurin or gilteritinib, FLT3 inhibitors that induce downregulation of MCL-1 to increase venetoclax activity (99); CUDC-907, a dual PI3K and histone deacetylase inhibitor that downregulates MCL-1, upregulates BIM, and induces DNA damage (100); MEK inhibitors (101); MDM2 inhibitors (102); PI3K inhibitors (103); and selinexor, an XPO1-selective inhibitor (104). In addition, an inhibitor of the Nedd8-activating enzyme (MLN4924) can upregulate Noxa, and 3-hydroxy-3-methylglutaryl coenzyme A reductase (HMGCR) inhibitors (statins) can upregulate PUMA. These two pro-apoptotic proteins, NOXA and PUMA, can neutralize Mcl-1 to boost the activity of venetoclax (105, 106). Ibrutinib, a Burton’s tyrosine kinase inhibitor, and ArQule 531, a multi-kinase inhibitor of Src family kinases and Burton’s tyrosine kinase, can also synergize with venetoclax (107, 108). Pharmacologic inhibition of mitochondrial protein synthesis with antibiotics that target the ribosome, including tedizolid and doxycycline, can potently reverse venetoclax resistance. Thus, inhibition of mitochondrial translation may be a new approach to overcoming venetoclax resistance (109). Owing to these preclinical results, an increasing number of clinical trials are ongoing.

## Predictors of Response to Venetoclax

The specific population that is most suitable for venetoclax remains unknown. However, recent studies have shown that mutations in NPM1, RAD21, MLL, or IDH1/IDH2 may predict venetoclax sensitivity (110, 111). In addition, a FLT3 internal tandem duplication gain or TP53 loss confers cross-resistance to both venetoclax and cytotoxicity-based therapies (112). According to a retrospective analysis, HMA plus venetoclax produces better results than traditional standard-of-care regimens in older patients with NPM1+ AML (113). To date, an increasing number of studies have shown that tumor cells with an NPM1 or IDH1/IDH2 mutation may more sensitive to venetoclax. Regarding other mutations, including FLT3 and TP53, VEN-based therapy may be a more effective therapy than traditional chemotherapy. Furthermore, blast cells of FAB M0/1 AML show higher sensitivity to venetoclax, while differentiated monocytic cells abundantly present in M4/5 subtypes show resistance to BCL-2 inhibition (114, 115). This may be associated with decreased expression of BCL-2 and a reliance on MCL-1 to mediate oxidative phosphorylation and survival. Therefore, as the number of patients treated with venetoclax increases, precise predictors of the response will be revealed.

## Conclusions and Prospects

Targeting the BCL-2 protein has shown compelling clinical promise in AML. Venetoclax, a selective BCL-2 inhibitor that was approved by the FDA for treatment-naïve elderly AML patients, has shown potential to be further explored. According to a recent study, previously untreated patients aged 75 years or older who were ineligible for intensive chemotherapy experienced a longer overall survival and a higher incidence of remission after treatment with azacitidine plus venetoclax than patients who received azacitidine alone (116). In addition, venetoclax+HMA has also been confirmed to be safe and effective in younger patients (117). Based on the encouraging results achieved with venetoclax, the scope of application of venetoclax is expected to be expanded in the near future for patients who are 75 years of age or older who are not suitable for standard chemotherapy, and potentially even for younger patients. In our center, we administer this treatment to elderly patients and even considered administering it to some young patients who are sensitive to venetoclax in a previous study. Of course, more standardized and larger clinical trials are needed to confirm the safety and effectiveness of VEN in younger patients.

Chimeric antigen receptor T cells, which have shown exciting results in B cell lymphocytic leukemia, are another a novel strategy for AML treatment. What outcomes would occur if this or other immune therapies was combined with venetoclax? Moreover, venetoclax-based therapies have shown effects on some high-risk AML subtypes. Could these therapies be combined with allogeneic HSCT or be used as a consolidation therapy after HSCT? Will these therapies affect graft versus-host disease or graft anti-leukemia? All of these questions remain unanswered. Furthermore, preclinical studies with MCL-1 inhibitors have demonstrated effective antitumor activity in AML. Will MCL-1 inhibitors be another promising strategy to cure AML? In summary, continued efforts to explore BCL-2 family proteins are necessary.

## Author Contributions

MZ designed the research. YW, YC, RS, LC, XX, XJ, XH and WL performed the research and analyzed the data. YW wrote the manuscript. All authors contributed to the article and approved the submitted version.

## Funding

This work was supported by grants from the National Natural Sciences Foundation of China (81970180; to MZ), the National Natural Sciences Foundation of China (81800105; to WL), and Tianjin First Central Hospital.

## Conflict of Interest

The authors declare that the research was conducted in the absence of any commercial or financial relationships that could be construed as a potential conflict of interest.
